# Spirit of the British and American Periodicals, &c.

**Published:** 1844-07-01

**Authors:** 


### Spirit of the British and American Periodicals.

### PATHOLOGY.

Observations on a Peculiar Nervous Affection incident to Travellers in Sicily and Southern Italy. By J. Hungerford Sealy, Esq. M.D. A.B. late resident Physician at Florence, Messina, &c. (Condensed from the Dublin Journal of Medical Science, May, 1844).

The author had frequent opportunities of witnessing the disease he is here about to depict.

It is characterised by an excessive irritability, attended with extraordinary mental and muscular activity, and seldom attacks the new comer, but more frequently those who have been resident between two and three years, and just beginning to suffer from *nostalgia*. There exists in it an inexpressible consciousness of disease, the mind is disturbed by visions; the imagination is morbidly awakened, yet the judgment still possesses its control over the mind, with scarce a capability of obeying its dictates.

Dr. Sealy is satisfied that it is a disease of climate. The modifications of it are great, and its grades various, from slight excitability to serious and formidable disease, affecting mind and body. According to the Doctor 'it seems a hyper-elimination of the nervous principle, a peculiar elastic evaporation, of a spiritual consciousness and capability, aroused by electrical agency or invisible atmospheric influence." The imagination and sanguineo-nervous temperaments are particularly liable to it, and suffer much during the prevalence of the Scirocco-wind, especially at Rome and Palermo, and at Naples and Sicily, when the atmosphere is charged with electricity. That all should feel excitement in that elastic atmosphere is not to be wondered at—it is when such excitement becomes excessive and permanent that it requires control. The extraordinary rarity of the atmosphere contributes much to this, the force with which impressions are conveyed to the senses. In Sicily the air is so attenuated and transparent that distance seems almost annihilated and sounds come on the air with appalling force. Some parts of Italy are found to possess this exciting influence more than others.

Whilst residing at Florence several cases of this nervous affection presented themselves to Dr. S. affording curious, and some of them most amusing traits. The severest case of it ever witnessed by him was in Messina in Sicily. On his arrival at Messina, from Naples, he was waited on by a gentleman stating that their resident clergyman was dangerously ill and requested his immediate attendance; he stated that the town was in a ferment about him, the Church of England service having been suspended for some weeks. Dr. S. immediately waited on his patient—he found him in bed—countenance haggard—eyes glaring out of his head and deeply suffused and bilious; skin dry and parched, and almost verging on the icteroid tint—tongue dry and red at edges, and covered with a brown fur in centre and back portion—pulse small and quick,—his general expression denoted the deepest misery, though his mind was perfectly clear—he had been ill three weeks. He had been under the care of a Sicilian physician, and had taken very little medicine, none of a purgative kind, though he felt he wanted it, as his bowels had not been moved for some days. The Sicilian doctor declared his complaint to be March fever, and was treating him accordingly with quinine—the only other medicine he had taken was an infusion of taraxacum, the Sicilian panacea for all diseases. Dr. S. advised blue pill in a smart dose, combined with compound colocynth pill to excite the biliary secretion—to this were added leeches to the head—mustard sinapisms to the feet—the pills to be followed up by a bitter saline mixture to full purging. After 12 hours there was a perceptible improvement—the patient had been well purged—his mind became more tranquil, and his nervous system much quieted. During the progress of his disease, his mental hallucinations were extraordinary, almost amounting to what the French Mesmerisers denominate *clairvoyance*, and his visions were frightful—his pervading wish was to tear everything near him, to shout, to sing and *curse*—he fancied he saw his limbs leave his body—he was convinced of the unreality of the vision, and of its being the result of a diseased imagination—yet so palpable was the delusive vision that he could scarcely correct the delusion by the utmost effort of his reason.

The bodily disease, separated from the mental hallucination, evidently had its origin in the biliary and chylopoietic viscera—this was indicated by all the symptoms as well as by the alvine discharges. This was the disease in its severest type. The minor modifications of the disease, met elsewhere, were not attended with such severe constitutional symptoms, and in many cases, where severe and distressing mental hallucinations existed, were unaccompanied by morbid appearances. Dr. Sealy states that he could almost always trace the disease to some engorgement of the *chylopoietic viscera*—he considered the disease as a modification of hypochondriasis, the nervous system being over-exerted by atmospheric influence, while the biliary and digestive systems were deranged at the same time.

The most successful treatment, according to the Doctor, is a modification of mercurial and vegetable purgatives, with a modified anodyne and stimulating plan of treatment.

---

### CASE OF DEATH FOLLOWING THE EXCESSIVE USE OF ARDENT SPIRITS; IN WHICH APPEARANCES SIMULATING IRRITANT POISONING WERE FOUND ON DISSECTION. By JOHN INGLIS NICOL, M.D. Inverness. (From the London and Edinburgh Monthly Journal, June, 1844.)

J. F. a young man about 26 years of age, had indulged in drinking a considerable quantity of whiskey in company with some friends. On his return home, two of his companions accompanied him, as he seemed much intoxicated. They had walked but a short way up an acclivity, when he fell, uttered a few words, and immediately became quite insensible. His companions, supposing him merely to be very drunk, carried him home, and after very considerable difficulty they succeeded in placing him in his bed. They could not say whether he was drunk at the time; they only thought him to be drunk. He was found dead next morning.

Seven hours after he had been discovered in this state, a post mortem was instituted—a few scratches on left cheek—back and depending parts of the body very livid; and under the angles of the jaws and along the sides of the neck, the colour amounted to a deep purple.

Thoracic viscera healthy. In the abdomen the distention of the larger gastric and gastro-epiploic veins attracted immediate notice—liver congested. On opening stomach, there was an evident odour of ardent spirits. The œsophagus, stomach, and a large portion of the intestines being opened to view, the whole of the mucous membrane, from about half-way up the œsophagus, along the whole extent of the stomach and for about 18 inches along the intestines, was found highly injected. The vascularity of the corrugated part of stomach was of a deep crimson colour—œsophagus and intestines presented a like appearance. Whilst the head was being examined, a large quantity of liquid blood flowed from the divided vessels; apoplectic congestion of the vessels of the investing membranes was very evident. The opinion given was that death was caused by apoplexy. After a careful examination not a trace of irritant or corrosive poison could be detected. Dr. Yellowly has given a paper published in the fourth volume of the Transactions of the *London Medical and Chirurgical Society*, from which it would appear that such cases as the present are of more frequent occurrence than is generally supposed.

---

### Solitary Confinement.

The effects of this system of prison discipline on mental and corporeal health, can only be determined by time and careful statistical records. The fifteenth Report of the Pennslyvania State Penitentiary lies before us, and is a very important document. In that establishment the silent and solitary system is carried out with great punctuality, and is under the care of an experienced physician, Dr. Hartshorne. It appears that the whole number of prisoners, during the preceding year (from March, 1843, to March, 1844) was 487, of whom, 325 were whites, and 162 coloured. The mortality was 11—5 white and 6 coloured. The per centage of mortality was therefore 1·53 as to the whites, and 3·66 as to the coloured. This shews a remarkable difference. During that year, 60 white prisoners and 23 coloured, were admitted in good health—and 53 white and 20 coloured, in imperfect health.

These facts proclaim no unusual mortality among the prisoners; and when we consider the habits of such persons, prior to imprisonment, the proportion of deaths is remarkably small—it is as nearly as possible the same as that of the citizens generally. The hygienic means employed are merely cleanliness of person and cell—free ventilation—good bedding—regular exercise in the cell yards—moderate diet—comfortable temperature—good cloathing, and constant occupation. Flannels next the skin have been found very beneficial in preventing rheumatisms, neuralgiæ, and coughs. Acute diseases have been few and of short duration. The chronic diseases have been chiefly those of the digestive organs, rheumatism, and venereal affections—occasionally scrofula and phthisis. The latter diseases rare among the whites. Dr. Hartshorne declares that he has not been able to discover any disease peculiar to the Penitentiary. The amount of tubercular disease, says he, has been much less than is generally imagined. In respect to the effects of this system on the mental functions, the physician avers that, from what he has seen, he has no reason to believe that it has any tendency to produce mental derangement in a person not constitutionally predisposed to that complaint.

"Instead of stultifying the intellect, as some imagine, I am fully satisfied that separate imprisonment is more apt to produce the opposite effect. The perceptions are evidently rendered more acute by continued exertion under the difficulties of restraint. The reflective powers also are increased by their unwonted activity under the exigencies of a seclusion, uninterrupted except by intercourse with intelligent and considerate men.

"The acuteness of perception among criminals is proverbial in all prisons; and the experience of this one long since proved that these faculties are by no means blunted there. Nor are the statements in regard to the subdued temper, and improved habits of reflection among separate convicts, mere idle assertions; as all those who have watched the mental developments in this place must be prepared to admit."

It can hardly be doubted that the silent and solitary system of prison discipline is that which most effectually corrects evil habits; and if, as these documents seem to prove, it is not injurious to health and life, the popular clamour against it goes for nothing.

### TWO CASES OF SCIRRHUS OF THE PANCREAS, WITH OBSERVATIONS ON THE DIAGNOSIS OF AFFECTIONS OF THAT GLAND. By F. BATTERSBY, F.R.C.S.I., &c. &c. (Condensed from the Dublin Medical Journal, May, 1844.)

Dr. Battersby was requested to visit Mrs. M., aged between 55 and 60, in Sept. 1843. She had enjoyed good health until two years previously, when she became subject to severe pains in the back, shoulders, and arms; these were supposed rheumatic. After a year there was discovered in the epigastric region a deep-seated pulsating tumor, size and shape of an orange, having a regular diastolic enlargement synchronous with the pulse, and a well-marked bruit de soufflet. The diagnosis then formed was *aneurysm of the aorta.* She also suffered from fluid eructations, and an obscure deep-seated pain. After a month, the tumor and pulsation disappeared—a new set of symptoms set in—scil. uneasiness over abdomen—stools passed with pain and forcing—great emaciation —complexion dingy and leaden—conjunctiva jaundiced—pain in abdomen, which was now prominent and tympanitic, particularly in the region of the cæcum—marked fulness in epigastric region, in which was felt a deep-seated, solid, and fixed induration. It had no pulsation, but a bruit de soufflet over it in the course of the aorta. Constipation aggravated her suffering, and bowels seldom moved without lavements and aperients—passage of stools very distressing and painful—stools consisted of watery, ropy mucus, deficient in bile. When solid, the fæces were not thicker than a small-sized finger. The body always bent forwards—no vomiting at any period—occasional eructations of a clear, watery fluid in small quantity, which was bitter—mouth always full of saliva—tongue pale and clean—no thirst—pulse 70, intermitting and variable—legs and thighs anasarcous.—Died Oct. 2d. Opiates and mild medicines were the only ones used. The ptyalism and eructations continued to the last. She always referred to the epigastrium as the source of all her suffering. The principal post-mortem appearances were, anasarca and ascites—large intestine very much contracted—great induration and thickening of the membranous structures within the abdomen: stomach very small, and its mucous membrane dark-coloured—its cardiac orifice much contracted by thickening and hardening of the cellular tissue around it—it was connected with the left extremity of the pancreas, which was universally hard and enlarged, and had lost every trace of its natural structure—near its centre was a thin, horny cyst, the size of a walnut; it lay over the aorta—its base was surrounded by a scirrhous formation. The inferior transverse portion of the duodenum adhered closely to the morbid pancreas, and was by it much contracted.

Here the pancreas was obviously the origin of a scirrhous affection, which, by extending to other parts, produced contraction of colon and of cardiac orifice of the stomach. Of all the usual symptoms of scirrhous pancreas, the only permanent ones present here were the epigastric induration and the emaciation—the others, scil. vomiting, jaundice, and epigastric pain, were either absent or obscured by more urgent symptoms, arising from contraction of the large intestines. Andral mistook a case for aneurysm of the aorta in a woman 54 years old, who complained of violent pains in the dorsal region, radiating to the left side of thorax; these pains affected the entire abdomen, and were lost in the region of the spleen. At the post-mortem, an enormous tumor was found in the position of the pancreas. Jaundice and vomiting are frequent attendants on scirrhous pancreas—they arise, the first, from obstruction or obliteration of the common or biliary ducts by pressure of the morbid growth, then seated in the head of the gland, which in the same way is often found to narrow very much the pylorus or duodenum. To compression of the vena portæ and cava we are to attribute the common occurrence of ascites and anasarca in organic affections of the pancreas. The great emaciation observed in scirrhus of the pancreas is explained by the great influence which diseases of this organ exercise over the functions of digestion and assimilation. Dr. B. next directs attention to one of the symptoms observed in the above case, viz. the ptyalism. He here gives numerous cases and authorities to prove that a reciprocal influence subsists between the pancreas and salivary glands. Fourcroy has observed, that, in obstructions of the pancreas, the salivary glands separated more saliva than usual. This is referred to the effect of sympathy. Certain instances of metastasis of inflammation are attributed to this sympathy. Augmented pancreatic secretion has been observed to be followed by a certain form of diarrhœa, and this in connexion with the salivary glands. While diarrhœa alternating with salivation is a consequence of inflammatory derangements of the pancreas, in scirrhus of the gland, on the contrary, continued constipation is almost always present, and is attended by salivation; which, coming on at a more advanced stage of the disease, is a symptom almost constant. This "balancement" of the secretion of the buccal and abdominal salivary glands is further illustrated by what occurs during pregnancy, in which salivation accompanied by constipation, is very frequent. The diarrhœa without fever, of pregnancy, in which the stools contain a great quantity of serosity, may have its origin in augmented pancreatic secretion. Hence we see the close relations and intimate sympathy existing between the abdominal and buccal salivary glands. Dr. Battersby refers cases of pyrosis with rejection of limpid fluid, alternating with diarrhœa, to chronic alterations of the pancreas—in this view he is supported by Andral.

---

ON TUBERCULAR LEPROSY. By A. S. SKENE, Esq. (From the London Medical Gazette, June 14, 1844.)

Frances Savoy, aged 46, a married woman, with a family of six children.—March 28th. She presented the following appearance:—The whole surface of the skin seemed as if smeared with oil. The entire face studded with tubercles varying in size from that of a pepper-corn to that of a bean—the larger ones, about the lower part of the face, enlarged the lips and cheeks. Nose much enlarged; alæ pendulous, with ulceration and thickening of the mucous membrane—eye-brows devoid of hair, cilia nearly gone. The whole of the inside of mouth and fauces studded with tubercles of various sizes—voice husky and nasal. Breath very fætid—many tubercles on the upper and lower extremities—respiration short—cough—sleeps and eats well—has little or no pain. Disease commenced about 4½ years ago. Her husband sleeps with her, and is in perfect health. One of her boys, eight years of age, has the disease.

Tranquil Robichaeux, a boy aged 15. March 29, 1844, he presented the following appearances:—

Entire face much swollen, and surface of skin as if smeared over with oil—studded with tubercles, very large, about the lower part of the cheeks and lips. Tip of the nose pendulous, and occupied by a livid, shining tubercle—lips much enlarged, full of tubercles on the inside. Tongue thickened, and protruding between the lips—palate and fauces thickly covered with tubercles. Uvula and tonsils tuberculated; one mass of disease, in fact, extending down into the pharynx. Cough—breath very fætid—voice a mere whisper—no hair or brows or eyelids. Calf of left leg and the right instep ulcerated—incipient ulceration at the roots of the nails—tumors in either groin—four large ulcers on left thigh. Sensibility of the diseased parts much impaired. Sleeps indifferently—appetite good. Disease commenced about six years ago. His uncle died of the disease in a shocking state about a month ago.

This disease, as described in the above cases, is identical with the tubercular leprosy which prevailed throughout Europe during the Middle-ages. The new locality for it forms a part of the province of New Brunswick. It is chiefly confined to the East side of the land lying between the bay of Chaleur and the estuary of the Miramichi River, and more particularly to the Settlements on the Neguac and Tracadie Rivers. No positive information could be obtained regarding the original appearance of the disease in this quarter: all that could be known was, that the first case occurred in the year 1817, in a woman named Ursale Landré, one of a family of 19 children. This woman married and came to reside in Tracadie. She had five children. After the birth of her youngest child, she fell into a bad state of health, and continued so for six or eight years. Blotches appeared on her face, extending over the upper part of the trunk and extremities. After some time, distinct lumps came on the face and inside of lips and throat—hair of eye-brows and eye-lashes disappeared—voice became hoarse. She died in 1829. Her husband died of it in two years after.

The points still at issue, connected with this disease, relate to,—1st, its peculiar nature; 2nd, the pathology; 3rd, the causes; 4th, the diagnosis; 5th, the prognosis, and 6th, the treatment. With respect to the first point, the primary cause of all the symptoms seems to be a morbid principle, *sui generis*, known only by its effects, which most probably resides in the blood—the most prominent symptoms being a perversion of nutrition, with a secretion of new products in the tissues of organs. The extrinsic causes cited by authors are: indigence, including filth, exposure to extreme temperatures, scanty or unwholesome food, with miasmata generated in the soil or subsoil. With respect to the other points, nothing either definite or satisfactory has been ascertained.

---

### ILEO-CŒCAL OBSTRUCTION.

The following fatal case of this disease was that of a medical student in Philadelphia, under the care of Dr. Dunglison.

Dr. Southwick, on the 18th of March, had an attack of bilious colic, with pain, flatulence, and bilious vomiting. Warm fomentations and opiates were prescribed, with enemata. 19th. Pain and vomiting abated—some meteorism and tenderness of abdomen—Seidlitz powder and castor-oil. Pulse natural. No evacuation. 20th. Castor-oil and ol. terebinth. Dr. Dunglison called in, and diagnosticated subacute peritonitis with cœcal obstruction. Ordered ten grains of calomel and two and a half of opium immediately—turpentine and castor-oil injections, and if no evacuations before next morning, to have 20 leeches applied. 21st. No evacuations—great decrease of the tenderness and meteorism. Twenty-five leeches over the ileo-cœcal region—castor oil mixture every two hours. In the evening a warm bath. 22nd. At two o'clock in the morning a profuse fluid fæcal evacuation, followed by several others—vomited several times—great dulness over the ileo-cœcal region—" death-like sickness." 23rd. Vomited a large quantity of fluid matters, with stercoracious odour. 24th. Pulse rose to 125—150—160, with great restlessness and anxiety. We need not pursue the details of treatment. He died at four o'clock of this day.

On dissection, the whole abdomen was tympanitic. A quantity of fæcal matter was found extravasated. Universal marks of inflammation were everywhere visible, with agglutination, and effusion. There was a binding down of the lower portion of the ileum, causing an obstruction there, and the appendix cœci had burst and discharged fæces into the general cavity.

We fear that the slowness of the pulse prevented early depletion in the above case. These abdominal, and especially cœcal inflammations, are equally insidious and dangerous.—*Medical Examiner, April,* 1844.

### Internal Aneurisms.

In the Philadelphia "Medical Examiner," of April 20, 1844, three cases of internal aneurism are related, of which we shall notice two.

*Case* 1.—Was a female aged 34 years, a hard-working woman and intemperate. She entered hospital, Dec. 28, 1843, presenting dyspnœa, especially increased about three o'clock every morning, relieved by expectoration. She had a harassing cough and much expectoration. Auscultation revealed the "*cantus avium omnium*" of Lænnec, that often shews a combination of bronchitis and empyema. She had little fever, increase of pulse, or pain. Much dulness about the region of the heart, with a roughened first sound. Her history threw no light on the nature of the malady. On the 15th January pneumonia took place in the lower lobe of the right lung, which terminated fatally in two days.

*Post Mortem.*—There appeared an aneurismal tumour, two inches in diameter, issuing from the arch of the aorta, which had not burst, but was filled with laminated coagula. The coats of the aorta were much thickened about its origin, with depositions obstructing the orifice of the left carotid artery. The aneurism was closely adherent to the œsophagus, and pressed on the trachea. One of the mitral valves was atrophied.

*Case* 2.—A female cook, aged 40 years, entered hospital, 26th January, 1844, complaining of cough, expectoration frothy—pain in the left axilla. Had cough for three months, but only kept her bed during the last fortnight, though her breathing was short for six years. Percussion clear on *left* side, but dull on the *right*. The breathing cannot be heard in the upper part of the right lung, except by great exertion of the patient. The resonance of the voice there approached bronchophony. The heart itself did not present any particular phenomenon. At the junction of the second rib with the sternum, there was distinct pulsation and heaving, together with some elevation of the part, and the sounds of the heart were here (right side) as audible as in the region of the heart itself. No aneurismal thrill could be heard or felt. January 31, she was seized with a great difficulty of breathing, and next morning was in a distressing state, and spat much blood and mucus. She lingered till the 4th February, when she expired.

*Post Mortem.*—Right lung compressed, and its space occupied by a large tumour caused by effused blood from an aneurism of the arch of the aorta, which had given way. The arch itself was much dilated, and lined with scales of bone.

We have a patient under our care at the present time who has suffered much from cough and dyspnœa, and in whom we recognize a distinct pulsation under the right clavicle. We have little doubt of an aneurism of the aorta.

---

### Observations on the Climacteric Disease, with Cases. By Henry Kennedy, M.B. &c. &c. One of the Medical Officers of St. Thomas's Dispensary.

[Dublin Journal, May 1844.]

It is now more than twenty years since Sir Henry Halford published his Paper on the Climacteric Disease, and for many a long year we have directed our attention to the malady in question, but not with the most satisfactory results. We occasionally see people, at all ages, droop in general health, but without any tangible or appreciable disease of any particular organ—exhibiting a declension of all the powers of life—a deterioration of all the vital functions—and this state continuing for many months, ending in death, or a gradual restoration to health.

Generally, however, some one organ, as the heart, the lungs, the kidneys, or the stomach gives way before the fatal termination arrives, and appears to be the immediate cause of death. That such a break-up of the constitution takes place more frequently at 63, than at 53—43, or 33, might be naturally expected; but beyond this, we verily believe that there is nothing about the "GRAND CLIMACTERIC" except the force of imagination. Dr. Kennedy believes that the malady in question is a substantive disease; but that it is not confined to the climacteric period, as he has seen it not unfrequently between the age of twenty and thirty years. But this is not all. Our author observes :—"*Were I to speak from my own experience, I should say that the persons who pass through life without having laboured under it, once, if not twice, are the exceptions to the general rule.*" The following sketch will shew that the climacteric disease is just what is familiarly called being "out of health," or "out of condition," for a longer or shorter period.

"Climacteric disease in general commences in a very gradual way. From three to six weeks may pass over, the individual not feeling quite well, and yet not making any distinct complaint. I have known it happen too, though it is not common, that the patient was observed by his friends to be looking ill for a considerable time before he made any complaints whatever. In the great majority of cases, however, the remarkable change the countenance undergoes is not observed till a later period of the disorder. Pains of one form or other are among the most common symptoms ushering in the attack: these may be of a darting and transient character, passing through the entire frame, or they may be more fixed and confined to a certain part. In the former case they are set down as rheumatic or gouty pains, according to the habits, or, it may be, the wishes of the individual; while in the latter nothing remarkable is to be observed about them, except that in general they are in a very marked degree periodic. Another very common symptom complained of in the earlier stages of the disorder is weakness, which is referred in general to the knees, the patient expressing himself in the usual way, by saying that these parts feel as weak as water. It is not alone when the patient is walking about that this weakness is complained of; on the contrary, they suffer from it while lying on a sofa, and I have seen it complained of to such a degree that it was described as amounting to absolute pain. It is also worthy of remark, in connexion with this sense of weakness, that it does not seem to be increased by any exercise the individual may take. In one instance, which will be given in detail, this symptom recurred for several days, and at a particular time of each day, before any other symptom showed itself, the individual during this period wondering what could be the cause of such weakness.

"It has been already stated that the disease under notice generally commences in a gradual way. To this, however, there are some remarkable exceptions, and this is an important point to keep in mind. I have known the disease commence with what may be called acute symptoms. Thus a common bilious attack has been followed at once by the usual symptoms, and well-marked climacteric disease has been established within fourteen days. A case of this sort will be detailed. A common cold, or influenza, have been already mentioned as ushering in the attack. But probably the most important of this class of cases is where the disease commences with head symptoms of so acute a character as to throw the medical man entirely off his guard. Under such circumstances a wrong view is very apt to be taken of the case, and erroneous treatment adopted in consequence. This will be alluded to again.

"After the pains, which have been before spoken of, have existed some time, other symptoms make their appearance, in quicker succession too than the commencement of the disease would lead one to expect. The appetite begins to fail; this soon increases to a total loss of it; and finally, should the attack be a well marked one, an utter aversion for all sorts of food succeeds. With this there is of course loss of flesh, and strength both of mind and body, and above all the sleep at this period goes astray. In a disease which is marked by such a variety of symptoms there is no more constant one than this loss of sleep; one exception only has come under my notice where it did not exist."

Thus the three chief symptoms are loss of flesh, alteration of countenance, and quickened circulation, with disordered function in one or more organs of the body. Or, to use the words of our author, the climacteric disease is—" a decay, for the time, of the several functions constituting life, but more particularly of those of the nervous system. To any one studying a case of the disease, it appears as if the system had got tired, and could not carry on its various functions with its wonted energy." If this be the climacteric disease, we agree with Dr. Kennedy, that it is to be met with at all periods of life, and in all classes of society—but more frequently, of course, in the later than in the earlier periods of existence.

In respect to treatment, it is hardly necessary to say that no specific plan can be laid down for a disease that has no specific character or seat. Debility is, no doubt, a prominent feature in the malady; but there is so often a local affection attending the general weakness, that we ought always to examine the head, chest, and abdomen. Such a state of bad health as is described by Dr. K. presents usually a considerable degree of derangement of the abdominal secretions; and if these are not corrected, tonics or stimulants will be of little avail. Alteratives, tonics, and change of air, are among the chief therapeutic agents.

---

On the Present State of Knowledge of the Nature of Inflammation. By T. Wharton Jones, F.R.S. (Condensed from the Medical Gazette, April 19, 1844.)

Retardation of the flow of blood in the small vessels with dilatation of their calibre, and at last stagnation of the blood-corpuscles in the vessels, constitute the first microscopical phenomena in the inflammatory process, as seen in the frog. There is good reason for thinking that the microscopical phenomena of inflammation are the same in man. It is generally supposed that the dilatation is primary, and the retardation of the flow of blood a physical effect of the preceding dilatation; the retardation however is greater than the dilatation will physically account for. With respect to the nature of the dilatation, it is now admitted that the dilatation of the arteries in inflammation is a state of relaxation or paralysis and not of activity. Mr. Jones, not being satisfied that the capillaries and radicles of the veins have contractile walls, which the small arteries are believed to have, and therefore unwilling to admit primary dilatation from relaxation in them, concludes that dilatation of the capillaries and radicles of the veins is secondary to the retardation of the flow of blood in the arteries, and is owing to distension from accumulation of blood. Henle thinks that relaxation and dilatation of the vessels, with retardation of the flow of blood, act in determining stagnation of the blood, and in this way; the retarded flow of blood, together with the relaxation and dilatation of the vessels, favours the exudation of serum; hence the plasma of the blood in the part becomes inspissated by a preponderance of protein matter over the salts. This inspissation of the plasma determines endosmotic changes in the red corpuscles, in consequence of which they are disposed to aggregate. Mr. Jones does not agree with Henle in this: he conceives that the stagnation of the blood must recognize some other cause than inspissation of the plasma. Mr. Jones considers that the proximate cause of inflammation, although affecting the constitution of the blood, does not reside in the blood only, but primarily in the agency on that fluid of the solids through which it passes in the capillary vessels—he thinks this appears from the limitation of inflammatory disease to a certain locality, from its easy reproduction at a subsequent period. The appearances, he says, attending the stagnation of the red corpuscles are such as might be supposed to be the effect of a suspension of the conditions by which, in the natural state, the red corpuscles keep in the middle of the stream, neither adhering to the walls of the vessels nor to each other, and not readily entering the smallest capillaries; the effect in fact of the establishment of an attraction between the red-corpuscles on the one hand and the walls of the vessels on the other, as well as among the red-corpuscles themselves, instead of the absence of attraction, or of the actual repulsion which naturally exists. Emmert, by way of explaining this attraction, indicates some of the conditions attending the operation of the attraction—he points out that constriction of the capillaries (small arteries) and the attraction between the parenchyma and blood-corpuscles are in antagonism—that when the constriction of the capillaries is *great*, the attraction between the parenchyma and blood is *small;* hence no congestion. When, on the contrary, there is *relaxation* and *dilatation* of the capillaries, the attraction is great between the parenchyma and the blood; and hence accumulation and stagnation of the red corpuscles.

Mr. Jones, before expounding his theory, claims the following postulates: 1. That the constriction and dilatation of the calibre of the small arteries at least, if not of the capillaries, is owing to contraction and relaxation of their walls in virtue of their contractility or tonicity, which is dependent on the nervous system. 2. That the ordinary tone of the vessels is determined by the moderate discharge of nervous influence. 3. That the relaxation, atony, or paralysis of the walls of the vessels on which their dilatation depends, is owing to the suspension of nervous influence. 4. That the relaxation with dilatation of the vessels from suspension of the nervous influence is the precursor of the stagnation. The theory which Henle has formed of the mode in which the exciting cause of inflammation determines the suspension of nervous influence is this: the exciting cause acts primarily on sensitive nerves, exalting their activity. The motor nerves of the vessels which have sympathetical relations with the excited sensitive nerves, are secondarily affected—this affection of the motor nerves which supervenes by reflex action on the excitement of the sensitive nerves, is one of depression, or suspension of action; of paralysis—this form of sympathy is called *antagonism.**

With respect to the inflammation of an organ occurring after section of some part of the sympathetic, Stilling refers it to paralysis of the walls of the vessels.

*Exudation.*—This commences immediately after or during the stagnation of the blood—it is at first serous, and afterwards pure plasma. So long as the vessels are entire, none of the corpuscles of the blood pass out or escape. With exudation is completed the inflammatory process, properly so called.

---

* There is scarcely a shadow of difference between this theory, and that propounded by Dr. Billing in his First Principles of Medicine.—*Rev.*

### PHYSIOLOGY.

### Muscle, a Neuromagnetic Apparatus.* (From the London Medical Gazette.)

The mechanism by which muscles contract or shorten their fibres is a question which has been much agitated. The view which I have taken is, that muscular fibre consists of a series of pieces in the form of discs, not immediately adherent to each other, but held together by an intervening substance, so yielding and elastic as to admit of very close approximation of the discs to each other, or their separation to a certain extent. These discs I consider analogous in their nature to *electro-magnets;* and I propose to call them *neuro-magnets.* The neuro-magnetic discs composing muscular fibre are of microscopical minuteness, and are arranged so as to operate on each other at very short distances only. The primitive fibrils of the nerves are disposed in loops across the muscular fibres, but not coiled round the neuro-magnets, as the galvanism conducting wire is around electro-magnets, when much electro-magnetic force is required. The nervous influence streaming along the primitive fibrils of the nerves, thus disposed in loops across the muscular fibres, communicates to the neuro-magnetic discs composing these fibres a magnetic state, by virtue of which they attract each other; this constitutes contraction of the muscular fibre.

---

### On the Relative Proportion of Centenarians, of Deaf and Dumb, of Blind and of Insane, in the Races of European and African Origin, as shown by the Censuses of the United States. By Dr. Forry. (From the New York Journal of Medicine, May 1844.)

In a preceding Number of the New York Journal of Medicine, Dr. Forry inserted an article, the object of which was to show the fallacy of an attempt made to prove, from the single fact that the census gives a much higher ratio of centenarians to our African than to our European race, that the two people constitute *distinct species,* and that their offspring, the mulatto, on the *assumed* ground of having a very short mean duration of life, is a *hybrid.* The subject of the relative number of deaf and dumb, blind and insane, in the two races, as well as the relative ratios among the slaves and free-coloured, was also noticed in the same article. In the Census of 1830 the number of deaf and dumb, and blind, was alone computed; that for 1840 included the insane also. In the Census of 1830 the ratio of whites, deaf and dumb, was 1 in 1964, and of the coloured population 1 in 3134; and of blind, the ratio of the former was 1 in 2651, and of the latter 1 in 1584. In this inverse result in the two races, the excess is about equal, the deaf and dumb whites being a little more than three to two, and the excess of coloured blind being in about the same ratio. According to the Census of 1840—

| | |
|---|---|
| The ratio of the white deaf and dumb was - - | 1 in 2123 |
| „ coloured „ - - | 1 in 2933 |
| The ratio of the white blind was - - - - - | 1 in 2821 |
| „ coloured „ - - - - - | 1 in 1509 |

---

* Extract from Mr. Wharton Jones's Lecture introductory to his course on Anatomy and Physiology at the Charing Cross Hospital, Oct. 1843.

| | | |
|---|---|---|
| The ratio of the white insane was | - - - - | 1 in 977 |
| „ coloured „ | - - - - - | 1 in 978 |

It is here seen that the proportions of the insane in the two races are identical, being about 1 in 1000. The discrepance among the States is so great as respects the ratios of the coloured insane, that no reliance can be placed on their correctness.

*Relative Proportion of Centenarians in the two Races.*—It has been proved by the Censuses that the coloured race, by virtue of some peculiar tenacity of life, far outnumbers the whites in centenarians. A comparison of the whites and the coloured race, at the age of 100 and upwards, in the Census of 1830, shows that the chances of attaining this great longevity are forty times as great with the free coloured as the whites, and with the slaves, eleven times as great; and in 1840, the disproportion, though somewhat diminished, shows nine slaves and thirty free-coloured centenarians to one white one. Dr. Forry maintains that this mere tenacity of life possesses not the least importance in the estimate of the mean duration of man's life. In the Report on the Sanatory Condition of the Labouring Population of Great Britain, it is observed that the greatest proportion of centenarians are of the labouring classes; and that instances of them have from time to time appeared amidst the crowded populations in some of the worst neighbourhoods of London, where the average duration of life is the lowest. Dr. Forry concludes that, as far as regards centenarians, they are to be found in the worst periods of man's history, as respects the condition of population, and often too, in the most insalubrious localities; that they abound in countries having a low mean duration of life, and that they decrease and finally disappear in proportion as the mean term of man's existence is extended by the improvement of his physical condition: and lastly, that they are found in that class of society, which has the lowest mean duration of life. He adds further, that the investigation of man's organization and of his natural history will continue still further to confirm the conclusion, that the entire human race have been made upon one model, and accordingly constitute but one family.

---

ON THE ACTION OF CONEIA ON THE BLOOD. By Professor HUNEFIELD. (From Simon's Beiträge zur Physiologischen und Pathologischen Chemie und Mikroscopie. 1844.)

As soon as any Coneia is mixed with the blood, a striking change became immediately perceptible. The blood was changed into a dirty reddish yellow greasy mass, which no longer allowed any blood-corpuscles to be recognized in it under the microscope. The Coneia employed was perfectly pure. On adding muriate of Coneia the blood remained unchanged. A short time since, I repeated these experiments with Coneia, and obtained the same result. The recent experiments directed to ascertain the causes of this re-action now show me, that Serum and Albumen coagulate in a similar manner; for if a glass rod, moistened with Coneia, be introduced into these fluids, a tenacious coagulum forms on the rod. Coneia shows this property with creosote, oil of turpentine, oil of bitter almonds and some other etherial oils, whilst others, as oil of rosemary, of peppermint, chamomile, valerian, petroleum and citron oils effect no coagulation in this way, and it is only on adding some water and shaking, that they produce something resembling a coagulum; differences which deserve to be more closely investigated. A moderately concentrated solution of dried blood soon undergoes a change of colour into a yellowish-brown, so that the fibrine of the blood becomes changed, and after longer standing, the blood becomes thickened.

Coneia, accordingly, deviates from the other alkaloids in these re-actions, and approximates, as was to be expected, to the etherial oils. Whether the other fluid alkaloids, as for instance, fluid Nicotine, produce a coagulating effect, I leave to be ascertained by those, who have those substances at hand. That Coneia, given by itself, and as a salt, kills with extreme rapidity, has been stated by Christison, and I have had an opportunity of ascertaining the great suddenness of its deadly operation. That the Salts of Coneia are equally poisonous, that a drop of Coneia can kill a large rabbit in a few minutes, that the blood-corpuscle, and the blood of the animal just killed, evince no change whatever; that death here occurs with peculiar nervous symptoms, whilst in the case of other narcotics it is ushered in with different symptoms, all these circumstances do not favour the opinion, that the actions of poisons are always of a chemical nature. A distinction may be made of poisons into blood-poisons and nervous-poisons. Whoever keeps all the physiological phenomena, namely, the great body of Nervous Physics, constantly before his eyes, will find that the actions of external powers on the organism cannot be explained on chemical principles, and that we are deeply indebted to some distinguished Physiologists, for their having given a check to the chemical views which are now becoming so prevalent. I let fall this observation, and reserve its extension for a more convenient time.

---

### Contributions to the Physiology of the Human Ovary. By Charles Ritchie, M.D. Glasgow. (From the London Medical Gazette May, 1844.)

1. The Fallopian tubes of the infant, and of the child before puberty, are perfect in their structure, although their patency is more or less obstructed at birth by the presence in them and in the Uterus of a tenacious glue-coloured mucus: and immediately before the establishment of menstruation, and throughout adult life, they are occupied, and have their uterine extremities in particular distended by a cream-looking fluid similar in appearance to the ordinary vaginal secretion, and which, as seen by the microscope, consists of innumerable very minute globules in masses of an oval form, differing from the globules of blood or pus.

2. The ovaries of new-born infants and children are occupied, sometimes numerously, by Graafian vesicles or ovisacs, which are highly vascular as early as the sixth year, and vary in size from the bulk of a coriander seed to that of a small raisin, in the 14th year, at which time they are filled with their usual transparent granular fluid, their contained ova can be detected, and their coats are so elastic, that their contents, on their rupture, can be projected to at least twelve inches: the existence of menstruation not being essential, therefore, to those conditions, either as a cause, or as an effect, and the possibility, even at this age, should rupture of the follicles occur, of their contained ova being conveyed to the uterus, along the Fallopian tubes, kept patent by their peculiar secretion.

3. The Graafian vesicles contained in the ovaries prior to menstruation, are found, as they also are in every other period of life, in continual progression towards the circumference of the glands, which they penetrate, discharging themselves by circular-shaped capillary pores in the peritoneal coat; the presence of the catamenia being thus no indispensable prerequisite to their rupture.

4. The establishment of menstruation, does not necessarily give rise to any immediate modification in the manner in which the ovisacs are discharged, or in their subsequent changes.

5. The ovisacs of the healthy menstruating female are, in general, larger and more injected with red-vessels, than they are previous to the setting up of that condition.

6. Menstruation gives rise to a congested state of the uterine vessels, as is manifested by the appearance.

7. The progression of the Graafian follicles, or ovisacs, towards the surface of the ovaries, their appearance under the peritoneal coat as copper-coloured macules, the absorption of that membrane, and of their own tunics, and the occurrence of a solution of their continuity at the point at which they unite, take place in the menstrual, just as in the anti-menstrual life.

8. The shape assumed by the ovisacs, depends on the part of the ovary they occupy.

9. The ovisacs of the human female do not require the presence of menstruation for their development or rupture.

10. The only circumstances essential to the discharge of ova, are the rise of the vesicles which contain them to the surface, and the gradual thinning of the ovarian peritoneum, and of the coats of the ovisacs at their point of union.

11. The appearances of the ovaries, Graafian vesicles, and of the blood contained in the latter, subsequent to their rupture, vary according to the time at which they are examined, and the absorbing power of the individual.

12. In case of the recent evacuation of an ovisac, the peritoneal coat of the ovary is occupied by a jagged slit or opening, having a florid vascular areola; in those of longer standing the opening is covered over, with the exception of a minute circular foramen in the centre, or, where the slit has been of great length, of two such openings, with new tissue, surrounded by claret-coloured margins.

13. The blood generally contained in the ruptured vesicles, is seen at first as a florid coagulum, next having its centre only scarlet-coloured, and its periphery more or less black: frequently the clot has a gamboge colour; lastly, the clot is found in different stages of absorption; sometimes in every variety of the uterine state the ruptured follicles are found empty, or containing only an aqueous fluid.

14. The coats of the ruptured ovisacs have been found in four different general conditions, apparently dependent on the relative degree of organisation, each class presenting also modifications of their respective characteristics.

15. The first class was distinguished by the attenuated state of the coats of the ruptured ovisac, and by the total absence of any organic changes in these, different from their condition previous to their discharge; the only alterations observable being—

A. The mechanical dying of their coats, of an inky black colour, proceeding from their contact with their contained decomposed blood.

B. The dying of their coats of a yellow tint, from their enclosed clot of blood having become converted into a rust-coloured pigment, not unlike some of the denser sputa of acute pneumonia.

This first class of ruptured ovisacs was found indifferently in all ages and states subsequent to puberty.

16. The second general class of ruptured vesicles was characterised, in addition to the presence of blood within their cavities, by organic changes in their coats—it was observed,—

A. Under the form of soft white bodies of a yellowish, fatty aspect, having the outer coat much thickened, their inner remaining as a delicate diaphanous pellicle.

B. As dense, white bodies of a whitish, shining, firm structure, their inner coat being the seat of these changes, and their outer adhering loosely as a transparent, pellicular layer.

A. The discharged ovisacs which constituted the soft white bodies were seen—

1st. At a very early period after their discharge, their walls distended with a coagulum, and painted with turgid vessels.

2. In a similar state, except that the inner membrane had a swollen, puffy look, and from the looseness of its adhesions might be easily gathered into folds.

3. At a period so advanced, that the clot had become uniformly black and solid, the inner layer was easily separated from the more external, which was also thickened and become yellow.

4. At a point still more advanced, the outer, thickened, yellowish coat had become corrugated into wrinkles or folds.

5. At a still more advanced period, the cysts were converted into yellowish white, and generally globular bodies.

17. These white bodies (corpora albida) were found in every variety of uterine condition subsequent to the establishment of menstruation, but never before it.

18. White bodies, as such, had no necessary connexion with the gravid condition.

19. The third general class of discharged ovisacs was distinguished by the secretion of an organized, yellow-coloured, brain-like, granular matter.

20. The fourth general state of the ruptured ovisac was peculiar to the impregnated and lactating female in the period between the eighth and thirteenth month after conception, and appeared to be a conversion of the forms already described, arising out of a higher and more perfect organization. Down to the seventh month of pregnancy the cysts contained in the ovaries did not differ in any respect from the cerebriform bodies found in the unimpregnated state, unless that they were sometimes plumper, more vascular, better developed.

21. The rose or red-coloured (corpora rubra) were not always present in the gravid or recently puerperal states.

22. The excess of corpora albida over every other physiological appearance in the impregnated ovary, seemed to result sometimes from the gradual obliteration of the vessels of the highly vascular outer coat, and cerebriform matter lining it.

23. In many puerperal women bodies of identical character, but in different stages of progression, were often found together, and it was then supposed that the best organised cysts were at once the most recent, and those which had furnished the fecundated ova.

24. In other pregnant women, in none of which had there been twins born, two bodies of a similar kind, and of equal organization, were found either in the same ovary, or in the two: and in such it was impossible to determine the productive from the non-productive vesicle, and their rupture was therefore believed to have been coetaneous, and that while the ovum of the one only had been fecundated, the coats of both, alike capable of taking on a higher organization, had been exposed equally to the increased ovarian circulation consequent on utero-gestation.

25. The simultaneous development in the ovaries of two corpora rubra was not met with in the present series of observations—a circumstance, which may be attributed, however, to the comparatively limited nature of the latter.

26. There was no evidence that granular matter was ever secreted around an unruptured Graafian follicle.

27. A nippled or cumulated protuberance of the surface of the ovaries over a granular cyst; crateriform openings on the apices of the latter, with vascular edges; and serrated "internal cicatrices," traversing the interior of the cephaloid bodies; and blood-vessels ramifying on their granular matter, were not peculiar to the gravid female.

28. Immediately on the arrest, and during the suspension, of menstruation, by any cause, the Graafian vesicles were found less vascular, thinner, and smaller, than their normal structure in menstrual life.

29. The co-existence of this modification of the ovisacs with the presence of white bodies in the ovaries, was not peculiar to the gravid state.

30. The obliteration of the cicatrices peculiar to menstruation, the presence of miliary ovisacs in the ovaries, and their appearance beneath the peritoneal coat as small copper-coloured spots, along with others of a yet more minute size, which had penetrated the substance of this membrane, and which did not require more than its thickness for their entire inclusion, together with the protrusion of some of these, often with extremely attenuated coats, in the form of very minute vesicles at the surface of the ovary, or along with puncta, or with a raw, blistered, or abraded appearance; and the existence, at the same time, in either ovary, of a cerebriform body.

31. The changes in the character and mode of the production and discharge of the Graafian vesicles which follow suppressed menstruation, from whatever cause the latter proceeds, was not seen to occur earlier than two months after the last menstrual flow; and previous to the expiry of this period, therefore, there were, in such cases at least as presented only the cephaloid bodies, no indications by which the fact of pregnancy could be determined, irrespective of the discovery of the ovum, and the greater organization of the deciduous fungiform villosities which are thrown out in the uterine cavity at every menstruation, and which, in such cases, suggest the idea of their being the rudimentary spiral arterial coils and horizontal sinuses of the membranous decidua.

34. The only special indication which distinguished the ovaries of lactating women delivered at longer periods than four months after the natural term, was the unusual presence in them of corpora albida.

35. The ovarian condition which most resembles what is here stated as occurring during lactation, is that met with in amenorrhœa from disease.

36. Graafian follicles of some size were found in the ovaries for fifteen, and white bodies for twenty years after the critical age, and the discharge of attenuated minute vesicles, apparently simple cells, from the surface of these glands, appeared to terminate only with life.

37. No relation was found to exist between the number of corpora albida discovered in the ovaries after death and that of the previous pregnancies; nor was there any specific distinction in the ovaries of women who had borne children, as compared with those of females who had not been gravid.

41. The uterine appendages were not, on every occasion, found remarkably injected with blood during or near to the time of the catamenial flow.

47. Ovisacs, containing altered blood, or having their coats dyed black (corpora nigra), or yellow (corpora lutea), may occur independently of menstruation, and were, exclusive of the effusion of lymph from the extremities of the tubes and the exterior of the ovaries, which appeared to have a similar origin, the only ovarian changes which could be suspected to have any connexion with venereal orgasm.

---

REMARKS ON THE EARLY CONDITION AND PROBABLE ORIGIN OF DOUBLE MONSTERS. By ALLEN THOMSON, M.D. &c. &c. (From the London and Edinburgh Monthly Journal of Medical Science, June, 1844.)

In the more important recent works on teratology, though anatomical structure and external form, are made the basis of division into classes, orders and genera, yet the place assigned to a considerable number of malformations in the divisions thus established, is regulated in some measure by the theoretical views entertained of the origin and nature of the malformation, founded upon known laws of embryology. Still even in the best of these classifications Dr. Thomson thinks that sufficient weight has not been given to theoretical considerations of the nature of malformations derived from embryology; and that, by due attention to these views, some improvement may be suggested, at least in the Section comprising malformation by excess. Thus, a careful revision of the various cases of redundant extremities appears to shew that there is not in them, as in most other malformations, a series of gradations from the least to the greatest degree of the abnormal condition, or from the mere superaddition of the joint of a finger to that of a whole extremity; but, on the contrary, that there is a sudden and wide hiatus between the form of polydactyle mal-formation, and the superaddition of a whole extremity. The organs which are usually, if not indeed alone, the seat of an increase in number in single redundant monsters belong to a particular class, those, viz. in which the component parts are naturally numerous, and which are observed to be most subject to undergo variations in number in the series of lower animals. Dr. Thomson is therefore disposed to think it more consistent with observed facts to consider as true double formations only those instances in which the parts of two cerebro-spinal axes, or of two vertebral columns are present, inasmuch as there is, in his opinion, no well-authenticated instance on record of the existence of the double condition of any important organ in which there is not reason to believe, that some degree of duplicity has also existed in the cerebro-spinal system. Dr. Thomson proposes to describe the early condition of double monsters, and to deduce, from a few observations made by himself and others, the most probable theory of their origin. He wishes, however, in the first place, to offer some remarks on the nature of the explanation afforded by a knowledge of embryology regarding the origin and nature of malformations in general.

With respect to malformations by defect, almost all teratologists are agreed in referring them to the occurrence of what has been called arrest of development. This theory, however, of arrest of development, is not applicable to all the malformations now under consideration. The various fissures or divisions of organs, as the cleft palate and hare-lip, open processus vaginalis or urachus, &c. are among the best examples of arrested development. But, again, many malformations occurring in single monsters, and all the forms of double malformation, receive little or no direct elucidation from this theory. During a part of the last century much controversy existed among scientific men as to whether mal-formations ought to be considered as dependent on an original tendency, or morbid condition of the germ, or whether they proceeded from injury or disease supervening during development. This controversy has now terminated, according to Dr. Thomson, in too exclusive a rejection of all accidental causes of malformation. Many malformations of the head and spine are with much probability attributable to the occurrence of hydrocephalus or hydrorachitis, at an early period of fœtal life.

Various attempts have been made to corroborate the mechanical or accidental theory of the origin of malformations by an appeal to experiments. Though the results of these experiments do not entirely establish the point, still they illustrate in some measure the extent to which some mutilations of the embryo may be indebted for their origin to mechanical causes acting during it's development. It must be admitted, however, that the constancy with which certain forms of malformation return, and the remarkable symmetry they very generally present, deprives this mechanical theory of all its probability, in reference at least to by far the greater number of them, and points to an original disposition of the ovum or germ. (The author promises a continuation of this very interesting article, of which we shall take care to present an analysis.)

---

### ON THE CHEMICAL PHENOMENA OF RESPIRATION. By M. GAY-LUSSAC, (Condensed from the Medical Gazette, May 10, 1844.)

The physiological world is divided between two opinions on the subject of the chemistry of respiration. According to one, propounded by Black, and advocated by Lavoisier, the formation of carbonic acid and of water takes place in the lungs themselves, and in virtue of the mediate contact of the oxygen of the inspired air with the blood contained in the delicate blood-vessels. According to the other view, the oxygen of the air does not act immediately on the blood in the capillaries of the lungs; it is merely absorbed there: the chemical phenomena in which it is influential take place without the lungs in the course of the general circulation; and it is only on the return of the blood to perform its lesser circuit that it gives off the products of the oxydizing processes that have already taken place in its mass.

The latter theory is chiefly indebted to M. Magnus for its development, and for the grounds upon which it was accepted in the world of science. The grand object of Magnus was to prove—1, the presence of carbonic acid in the blood; 2, the different proportions of carbonic acid, and of atmospherical air or oxygen in the blood of the arteries and veins. M. Gay-Lussac questions the validity of M. Magnus' conclusions; some of his inferences being found even in opposition to those which his facts would warrant. According to Magnus' theory, venous should contain more carbonic acid than arterial blood. Yet by actual experiment this chemist obtained less from venous than arterial blood. This grand contradiction, however, is not the only one. Both kinds of blood contain oxygen and azote as well as carbonic acid. If azote be formed in the course of the circulation, as experience has proved, the quantity of this gas ought to be less in arterial than in venous blood. Yet, according to the figures in Magnus' experiments, arterial blood contains half as much azote again as venous blood. The relative proportions of oxygen alone seem at first sight to agree with Magnus' theory, venous blood being found to contain about one-half less oxygen than arterial. Now it is obvious that the carbonic acid which makes its appearance during respiration, is formed at the expense of the oxygen absorbed by the blood; hence there must be a certain relation between the volumes of these two elastic fluids. If, for instance, we know the relative volume of the carbonic acid expired in a given interval, we know that the corresponding volume of oxygen absorbed must be at least equal to it. Unfortunately, the conclusions of M. Magnus regarding the quantities of carbonic acid and of oxygen discovered in arterial and in venous blood respectively, are mutually subversive of each other.

For want of positive data, which are not to be attained from the labours of Magnus, we shall take them from Davy. 1. Davy states that a man expires thirteen cubical inches of carbonic acid gas in one minute. 2. That the heart, at each pulse, expels one ounce of blood; and, taking the number of contractions at 75 per minute, 75 ounces, or 115.7 cubic inches of blood, will pass through it in the same interval of time. Now 115.7 cubic inches of blood having given off 13 cubic inches of carbonic acid, 100 of blood will contain 11.23 of this gas, a quantity which the blood might readily part with, as, according to M. Magnus' experiments, it contains much more than 20 per cent. of the gas. But, supposing that the venous blood gave off 11.23 per cent. of its volume of carbonic acid, it is evident that to produce this, arterial blood ought to contain at least an equal volume of oxygen, say 11.23. Moreover, as in the respiratory act, of four parts of oxygen absorbed, three go to form carbonic acid, and one to form water, the blood must have taken up, in its course through the lungs, not only 11.23 oxygen, but $11.23 + \frac{11.23}{3} = 14.97$, a quantity which is sixteen times greater than the 0.926 of oxygen which pure water can dissolve in the same circumstances, that is to say, in contact with atmospherical air. And further, if we allow with Magnus that venous blood, on reaching the lungs, still contains about one-half of the oxygen originally dissolved in the arterial blood, the whole quantity which the latter ought to contain at its exit from the heart will be, first, 14.97 destined to be used up in forming carbonic acid and water, plus 7.48 remaining in the venous blood; in all 22.45, a quantity which would require us to suppose that, in contact with an atmosphere of oxygen, 100 of arterial blood could take up $22.45 \times \frac{100}{21} = 106.9$, or more than its own volume of the gas. Such a capacity in arterial blood for dissolving oxygen, though not impossible, would require proof.

Magnus explains the change of colour in venous blood entirely by the loss of carbonic acid in the lungs. But it is not proved that venous blood exhales carbonic acid in the lungs; and then, if it were, the quantity of carbonic acid which it does not give off, according to Magnus, is still so great in comparison with what it does eliminate, that it appears impossible to explain by a difference in quantity relatively so small, any change of colour so remarkable. It is therefore obvious, that the theory of respiration, advocated by Magnus, has as yet no sure foundation, and that the chemical phenomena of this vital act require to be discussed anew. M. Gay-Lussac, with M. Magendie's able assistance, proposes to go over the ground of the theory of respiration.

---

On moving Molecules in the Interior of Cells. (From the Provincial Medical and Surgical Journal, June 5, 1844.)

In a letter from Mr. Addison, so favourably known to the medical profession for his numerous and valuable microscopical researches in anatomy, to Dr. Streeten, the editor of the Provincial Journal, the former gentleman states, among other matters, that he has discovered, by employing a magnifying power, from 700 to 1000 diameters linear, an essential distinction between cells having moving molecules visible in their interior, and a disposition to discharge or liberate them; and those which have apparently passed through or have been arrested in the progress of what appears to be a vital and normal change. The letter contains the result of a more minute and critical examination of the objects circulating in the blood of the part, or discharged in the frequent and unimportant affections of the skin and mucous membranes, with especial reference to these active, moving, or living molecules, so numerous within the blood, the saliva, and the pus-cells. The paper is accompanied by a drawing which gives a faithful representation of the appearances exhibited in the colourless blood and in the pus-cell—for further information we beg to refer to the original paper.

### MATERIA MEDICA AND PHARMACY.

### ON THE POISONOUS PROPERTIES OF THE BARK OF THE LABURNUM-TREE. By ROBERT CHRISTISON, M.D. (From the Edinburgh Medical and Surgical Journal.)

A farm servant, named Gordon, 18 years of age, being on bad terms with his fellow servant the cook, administered to her in some broth a portion of the bark of the *Cytisus Laburnum,* or common laburnum tree; this he did for the purpose of exciting vomiting. The cook soon became very ill, and in five minutes was attacked with violent vomiting. After the first attack of vomiting, which occurred at three in the afternoon, the retching and vomiting continued incessantly throughout the entire evening, night and subsequent day; there was at the same time shivering, general pain in the belly, especially in the stomach, and such feebleness from the moment she first took ill, that she with difficulty walked to her bed; severe purging also occurred on the morning of the second day. It was some days before she was able to resume her work. The sickness, vomiting and purging, however, continued to recur, in some degree, daily; pains throughout the abdomen; she rapidly fell off in looks, flesh, and strength; about six weeks after she first took ill, she was forced to give up service; her complaints went on without any intermission, except in degree for about seven months, when she was first visited by Dr. Ross, who was sent on the part of the law-authorities to investigate the particulars of the case, He found her labouring under symptoms of marked gastro-intestinal irritation, such as vomiting, especially after food, pains in the abdomen increased by pressure, diarrhœa with tenesmus and slightly sanguinolent stools, flatulent distension of the belly and the like. There was great debility, impaired appetite, hurried and laborious respiration, a rather frequent and easily excitable pulse, strong bellows' sound over the roots of the large vessels of the heart, a pale countenance, bloodless lips, and a pale, glazed tongue. The patient recovered very slowly. This woman having enjoyed robust health previous to the administration of the Laburnum-bark, it cannot be doubted, that if the bark caused the first symptoms, it must also have occasioned all the subsequent illness.

---

### ON ARSENIC AS A POISON; ITS TESTS AND ANTIDOTE. By E. J. SHEARMAN, M.D. Rotherham. Read before the Sheffield Medical Society, March 7, 1844. (Abridged from the Provincial Medical and Surgical Journal, April 3rd, 1844.)

In order to show the existence of arsenic in a court of justice in England, we should be able to prove the following facts so satisfactorily, that a *jury* may not only *see,* but *perfectly understand them.*

1st. The *metal* should be produced either from the contents of the stomach, intestines or urine, if the patient should survive; or, if dead, from these and some part of the body.

2nd. We should be able to prove that the animal substances experimented upon were the excretions, and parts of the patient's body only; *unmixed with any other matter.*

3rd. We must also prove that the tests we use to shew the existence of arsenic *have not a particle of arsenic in themselves;* and this requires great caution, because a skilful advocate might make a guilty prisoner appear innocent, owing to the omission.

4th. As *antimony, bismuth, tin, zinc, lead, tellurium, cadmium, selenium,* and *potassium,* sublime in a somewhat similar manner to *arsenic,* and *may be mistaken*

*for it;* it is quite necessary for an inexperienced eye to guard against such a mistake.

When the contents of the stomach, or a decoction of the stomach, liver, &c. in distilled water, are to be operated on, the most efficacious manner of proceeding is that recommended by Christison. The first point is to get rid of the animal matter. When a clear solution is obtained, with but a small portion of animal matter in it, *the ammoniacal nitrate of silver gives a lively lemon-yellow precipitate —the arsenite of silver.* The *ammoniacal sulphate of copper gives an apple or grass-green precipitate—the arsenite of copper.* And the transmission of *sulphuretted hydrogen gas* through this solution, previously neutralized, and then slightly acidulated with acetic acid, throws down an abundant *sulphur-yellow precipitate* —the *sulphuret of arsenic.*

It is the best plan to boil the last precipitate in the solution a few minutes, which drives off the excess of sulphuretted hydrogen gas, and allows the whole of the sulphuret of arsenic to deposit. This is to be collected in a filter; dried on a watch-glass; introduced into the bottom of a bulbed tube, and covered with black-flux, or, what is better, freshly-ignited charcoal. The tube is then to be heated by a spirit-lamp, first at the part just above where the flux is, and then gradually below, so as to sublime the metallic arsenic all around the tube; which forms a *brilliant polished metallic appearance,* that cannot be easily mistaken when once seen. The metallic arsenic should afterwards be oxidized by the heat of the spirit-lamp and the oxygen of the air, and driven up the tube, so as to allow it to form *octohedral crystals of arsenious acid, with triangular façettes.* The other mode of obtaining metallic arsenic is by Marsh's hydrogen test—the objection to this method is the necessity for using zinc, which itself often contains arsenic. The next method is that of Mr. Ellis of University College, London, for which we refer to the paper itself.

There is another test which Dr. Shearman thinks deserving of attention—viz. *the decomposition of distilled water by galvanism, to which the suspected solution is added, with pure sulphuric acid,* collecting the hydrogen from the negative pole, igniting it, and examining the stain left in a glass-tube open at both ends. If there is the least particle of arsenic, the hydrogen will combine with it, and you then have a stain of *metallic arsenic* with *rhomboidal crystals;* which you may *oxidize, collect and dissolve in water; go through the fluid tests, reduce the sulphuret in a tube, and sublime it into arsenious acid again.* This is the most delicate test.

The most likely substance to be mistaken for arsenic by any of these tests is *antimony,* because *antimony sublimes into the same kind of crystals as arsenic does.* Attention to the following rules will enable us to distinguish. *Metallic arsenic sublimes* at 356° *without liquefying into rhomboidal crystals; arsenious acid sublimes at* 380° *into octohedral crystals; metallic antimony sublimes* not under 810°; and cooling, acquires a highly lamellated texture, and yields *octohedral crystals like arsenic.* After you have obtained a deposit of the suspected metal in a glass tube, if you heat the tube gradually by a spirit lamp, *should the metal be arsenic,* it sublimes on the *cool part of the tube* into *octohedral crystals;* which can be *dissolved in water,* and tested by the three fluid tests. If it is *antimony,* it first produces *dense white fumes,* and an *amorphous white powder is deposited;* and the *heat being kept up,* the tube is lined with a *white crust,* insoluble in water. The question then comes—*how can a witness swear most positively that a substance is arsenic, and nothing else? and how can he convince an unscientific jury of that fact?* I think only thus: 1. *By producing the metal and showing its crystals.* 2. *Reducing it to the oxide and showing its crystals.* 3. *From these crystals going through all the fluid tests.* 4. Reducing the sulphuret again to its metallic state, then to the oxide, and again going through the fluid tests. 5. And if this be shown clearly, it will *be impossible for any advocate to mislead a jury.*

With respect to antidotes, Dr. Shearman has found the *moist hydrated peroxide*

*of iron* to be the most efficacious. A table spoonful to be given in plenty of water, every five minutes to an adult. *This is perfectly inert, if kept dry.*

---

I. LECTURE ON THE STATE OF PHARMACY IN ENGLAND, AND ITS IMPORTANCE TO THE PUBLIC, WITH REMARKS ON THE PHARMACEUTICAL SOCIETY. By J. LLOYD BULLOCK.
II. PHARMACEUTICAL JOURNAL AND TRANSACTIONS. Edited by JACOB BELL. June 1, 1844.

The object of this lecture of Mr. Bullock is to impress upon the minds of the rising generation of *Pharmaciens* the indispensable necessity of making themselves thoroughly acquainted with the principles and practice of Chemistry. In the course of the lecture, however, Mr. Bullock takes occasion to allude to certain acts and proceedings of the Pharmaceutical Society, of which he disapproved so very much, that he actually withdrew from that body. The reasons for his withdrawal he states in very plain, intelligible terms. In the first place he says that "the Society, if established for the benefit of Pharmacy, should have kept to a strictly honorable course." Mr. Bullock considered that the Council of the Pharmaceutical Society deviated from this honourable course, when they held out an expectation, that members of this Society only would be held legally qualified to pursue Pharmacy, whilst persons who should not become members would lose this important privilege. Now, really taking every thing into consideration, we cannot go all the way with the learned Lectuier in accusing the Council of having deviated from the "strictly honorable course," because, forsooth, among the other inducements to become members of the Society, one was made to consist in making people believe that the privilege of practising Pharmacy was to be conceded exclusively to those who should enrol themselves among its members. The Council were desirous that the Chemists and Druggists should do that which would raise their character and iespectability, and to induce them to do so, they held out a sugar-plum—

*ut pueris olim dant crustula blandi*

*Doctores, elementa velint ut discere prima.*

We think we might dispose of the other charges brought against the Society in a somewhat similar way. The Council of the Society, as men of sense and men who possessed a knowledge of the world, well knew that different individuals are induced to adopt the same measures by different motives—some must be coaxed, some frightened, some irritated into the adoption of certain plans; accordingly they very properly acted on the hint—

*Irritant, mulcent, falsis terroribus implent.*

With respect to the distribution of the diploma to the members of the Society, we can see no harm in it. We all know that a man likes to have something to look at, and to shew for his money. There is one charge made by Mr. Bullock, which we cannot at all understand; we mean that of obliging every member and associate to pay for the Pharmaceutical Journal, instead of permitting its transactions to be published in "the proper channels." We cannot, for the life of us, see a more proper channel than the Pharmaceutical Journal for the publication of pharmaceutical subjects, and we believe that, until the establishment of the present Pharmaceutical Journal, we had no other journal whatever exclusively devoted to that purpose.

### VETERINARY PHARMACY.

Veterinary Pharmacy, and indeed veterinary practice altogether, were, until some few years back, in a truly deplorable state. Analogy seems to have been the almost only guide in the selection and employment of horse-medicines formerly, it being the general belief that most medicines had a similar effect on horses and on the human subject. Mr. Bracy Clark, in his *Pharmacopœia Equina*, has shewn that many remedies in constant use for the human subject, have a very slight action, and, in some cases, no apparent action whatever, on the horse. Among the purgatives he enumerates jalap, elaterium, gamboge, and colocynth, as producing no laxative effects on the horse, although administered to such an extent as to occasion the death of the animal. Among the publications which have tended to promote the advancement of veterinary medicine we may notice the *Veterinarian*, commenced in 1828, by Mr. Percival and Mr. Youatt. This journal is intended to supply the place in the veterinary department, which is occupied by the Lancet and Gazette in the medical profession.

### THERAPEUTICS.

### On the Ready Curability of the more Acute Form of Hydrocephalus, in its Earliest Stage, under Active Treatment; with a Case. By Alexander Harvey, M.D. (From the Northern Journal of Medicine, June 1844.)

The writer, after stating the various opinions now generally entertained by the profession with respect to the degree in which hydrocephalus in its more acute form is amenable to remedies within the first two or three days from its outset, very justly observes that the settled convictions of our minds in regard either to the curability of the disease or the diagnosis of it, in its early stage, will very materially influence our conduct in this department of practice. And accordingly it will be found that those who entertain a confident persuasion of the ready curability of the disease, at the period already mentioned, and are impressed with the importance of being satisfied, in judging of the diagnosis, with indications much short of certainty, are at once prompt and energetic in their practice; that those who are more or less sceptical as to its curability, or require rather clear evidence of its existence, are so far influenced thereby, in cases at least which are at all doubtful, as to decline or defer any very active treatment; and that those who, though agreeing generally in the views of the former class, are less confident in their apprehension of them, and less sanguine as to the general efficacy of bold and energetic practice, are proportionally less prompt and decided in their treatment. The principle which guides Dr. Harvey in his treatment of this disease is, that hydrocephalus, in its more acute form and in its earliest stage, is readily amenable to active treatment, and that its diagnosis may in general be then made out with sufficient confidence to warrant such treatment. The evidence in favour of this principle he declines adducing for the present, but gives a case which is strongly illustrative of its truth. It was that of a youth, 14 years of age, one of a family of which three had died of hydrocephalus, and who himself was predisposed to the disease. This boy, after being confined to the house for a slight cold, began to complain of pain in one of the temples, with a quick and sharp pulse; for these he was briskly purged with temporary relief—the pain however returned with increased severity—restless nights—the pain darted through his head in twinges, and was greatly aggravated on moving—heat of head increased—he was bled to 12 oz. hair removed, and cold applications to head—again briskly purged—the pain of head still remaining, he was again bled—purging still kept up—the patient recovered.

In this case the inflammatory appearance of the blood drawn strengthened the suspicion that the case was one of inflammation within the head, in its first stage, a suspicion fully confirmed by the effects of treatment.

### Case of Death by Poisoning with the Leaves of Aconite or Monkshood, passed off in jest for Parsley. By Al. Ramsay, Surgeon. (Northern Journal of Medicine, June 1844.)

At about 11 a.m. a boy named Hanton, aged 14, on passing a garden in the parish of Murroes, looked over the wall, and asked a young man in the garden, "if he would give him some parsley"; to which it was answered, that he would get some new parsley, as being much better than the old kind. The young man accordingly gave Hanton a handful of green leaves, of which he ate some. In about two hours after he complained of a burning sensation in the mouth, throat, and stomach, and was very sick. For this his mother made him take a glass of whiskey. Some time after this he took a fit and fell on the ground—at 6 o'clock p.m. on his mother returning from her work, she found him lying across the bed, with his hands in his pockets, dead and stiffening. At the post-mortem the blood-vessels within the head were found enormously distended with dark-coloured fluid blood; upwards of a pound of which escaped from the skull and spinal canal. The stomach was empty, with a deep inflammatory blush over its whole internal surface and here and there patches of a darker colour—further dissection was not allowed.

Death appears in this case to have been caused by the apoplectic state of the brain, probably ending in asphyxia.

---

### Tincture of Iodine.

In the Montreal Medical Gazette, (April 1, 1844) Dr. Crawford draws the attention of the profession to the beneficial influence of Tincture of Iodine (Magendie's form), applied to variola, erysipelas, acute rheumatism, and neuralgia. During the last four years, erysipelas has been very prevalent in Montreal, and Dr. C. has applied the iodine with great apparent advantage. He does not exclude the use of constitutional remedies; but he has seen the most decided advantages result from the local application, where no internal means were employed. In variola he tried the comparative merits of the iodine and the nitrate of silver, but gives the preference to the former. This application is very manageable and very bearable.

---

### On the Moral Treatment of Insanity in Bethlem Hospital. By R. Willis, M.D. (From the Medical Gazette, May 3.)

From the Report* we learn that, in the course of the year 1843, 284 patients—109 males, 175 females—were admitted into the Royal Hospital of Bethlem, and that the relative number of cures, progressively on the increase, has been greater in this than in any preceding year; being as high as 56 per cent. upon the admissions. The advantages of providing employment for the patients have been ascertained; and to this circumstance doubtless much of the success of the remedial measures must be ascribed. Besides the ordinary and incidental occupations, as gardening, cleansing the wards, &c. a range of workshops has been added. Experience has fully proved that no risk whatever is incurred in allowing the patients the use of the ordinary implements necessary in their respective

---

* The General Report of the Royal Hospitals of Bridewell and Bethlem, and of the House of Occupations, for the year ending 31st December.

trades. On the female side, the matron has added to the old list of occupations straw-plaiting, bonnet-making, shirt-making, lace-making, and fancy-work, with the best effect. Considerable additions have been made to the amusements of the patients on both sides of the Hospital. On the male side a library is in course of formation, and contains a selection of useful and entertaining works, which have been under the charge of a patient as librarian. Chess, draughts, cards, and backgammon, have long been allowed with very beneficial effects. On the female side, a piano-forte has been purchased, which is a sonrce of great gratification both to those who can play, and to the other patients also. It was in Bethlem that the example was set in this country of liberating lunatics from personal restraint—in 1840 the weekly average number of patients under restraint was 13⅐; in 1841 it was 9; and in 1842 and 1843, about 3. The attendants are aroused to increased vigilance by the necessity of closer attention; and living with and among the patients, gradually acquire their confidence, and become the directors of their occupations, and the companions of their amusements. By the new regulations every patient has a tepid bath once a week, unless forbidden by the physician, and each patient has also two changes of body linen in the course of the week, instead of one as formerly. Considerable improvements have been made in the bedding, horse-hair and wool mattrasses having been substituted for the old straw sack. Tea and coffee with sugar, now form part of the ordinary dietary of the hospital. This change has of itself entailed an additional expense of £400. per annum.

The necessity of seeking early advice in cases of insanity is well known to the profession, and its advantages are illustrated in the Report in the best of all ways, an appeal to the success of the remedial treatment at different periods of the disease. The chance of recovery is in exact proportion to the shortness of time during which the disease has existed; the probability of effecting a cure of the patient who is received within one month is generally just double that of restoring one who is received within two months from the commencement of the attack. From the table No. 8, we see that of the entire number of 284 patients, 106 were received within one month; but between one and three months the number is still greater, viz. 108. The great relative disproportion of cures effected in males and females, is somewhat startling. Whilst 103 of the 175 female patients are discharged cured within the year; of the 109 male patients no more than 56 are so discharged. The cause of this immense disproportion is well worthy of consideration. The total proportion of cures, and the general sanatory state of the hospital, it is most gratifying to observe have gone on steadily improving. In the middle of the last century the cures amounted to only 33.20 per cent. annually, and the ratio of deaths was as high as 25.43. Last year (1843) the ratio of recoveries reached 56 per cent. and the deaths were only about 6 per cent.

Another point alluded to in the Report is the admission of pupils to the wards of Bethlem Hospital, as a school where they may acquire a competent knowledge of insanity.

---

### On the Use of Oleum Tiglii, externally applied, in Cases of Dysphonia, Cynanche Trachealis, &c. By S. Corp Ellis, M.D. (From the New York Journal of Medicine, May, 1844.)

In the case of a lady who for several weeks had been unable to speak, except in a faint whisper, Dr. Ellis directed her to rub three drops of croton oil along the trachea; a few hours after doing so, she had a sense of constriction about the upper part of the thorax, followed by "*two*" expectorations of sanguineous mucus. Shortly after, her voice became quite natural.

In a case of cynanche trachealis, in a child three years old, wherein the little patient lay in a comatose state, with his neck stretched back to its utmost extent, countenance livid, respiration nearly annihilated, Dr. Ellis ordered the following ointment: *scil.* twelve drops of croton oil with two drachms of lard. A portion, the size of a large pea, was directed to be gently rubbed along the course of the trachea every fifteen or twenty minutes—in about 15 minutes after the first application of the ointment there was a visible improvement; the breathing became easier, and the countenance assumed a more natural appearance, coma began to disappear, and a quantity of tenacious mucus was forcibly ejected. The use of the ointment was continued at much longer intervals; the next day the membrane disappeared entirely, and the child got well. The same treatment was adopted in other cases with similar success.

---

### ON THE USE OF MUSTARD IN THE CONVULSIONS OF CHILDREN. By CHAS. S. TRIPLER, M.D.

Dr. Tripler treated a case of convulsions from teething with mustard, which he employed for its emetic effects—he had previously employed antimony, sulphate of zinc, sulphate of copper and the usual emetics, but without being able to make any impression on the stomach. In a few minutes after its employment it arrested a fearful attack of convulsions, which had lasted five hours, and that without vomiting the patient for some time afterward—he afterwards used it in several other cases and with equal success. Its efficacy seemed to have no relation to its emetic properties.

(The Doctor should have stated the dose, mode of administration, &c.)—*Rev.*

---

### MIDWIFERY.

### A LIVING CHILD, 39 DAYS ATTER QUICKENING—147 DAYS AFTER CONCEPTION.

This curious case happened in the practice of Mr. C. Smythe, of Castle Douglas. A female, in her second pregnancy, and in the 147th day of utero-gestation, had a severe flooding, with rupture of the membranes. Labour did not take place till the next night, when a very small but well-formed fœtus was expelled, giving no other indication of life than a very feeble action of the heart, and a strong pulsation in the cord. By proper means, however, it was resuscitated, and cried as strongly as a child born at the full period of pregnancy. It weighed less than two pounds, and measured exactly twelve inches. It swallowed some nourishment, but died at twelve hours and-a-half after birth. The Membrana Pupillaris was entire—the testicles had not descended—the head well covered with hair. Some ecchymosis appeared on the back and on the hands before death. From peculiar circumstances, it was evident that the mother of the infant was perfectly correct in respect to dates.

There was clearly nothing in the organisation of this child to prevent its growing to the age of maturity. We believe there is a case recorded in the Edinburgh Medical and Surgical Journal, where a premature fœtus of eighteen weeks was preserved by great care. In the present case the period of utero-gestation extended to 21 weeks.—*Ed.*

### Inversio et Extirpatio Uteri. (Prov. Med. Journ.)

This case occurred in the practice of Dr. J. G. Crosse, the talented surgeon of Norwich. A female had a bad delivery, of her first child, at the age of 29 years. Two years afterwards, January 14, 1843, she fell in labour with profuse hæmorrhage. The child was safely born, but she continued very weak, and almost pulseless for many hours. The Placenta was delivered piecemeal. On using the catheter, 36 hours post partum, Dr. Crosse found the uterus inverted in the vagina, and used every effort, (in conjunction with the surgeon who had delivered her,) to replace the organ, but in vain. The tumor was firm and contracted. At the expiration of four days, the patient, by straining, forced the uterus through the labia into open day. The fundus had a ragged sloughy appearance. She continued in an alarming state, with the pulse at 120 or more. By various manipulations the tumor was supported, and the patient rendered more comfortable: but still the profuse mucous discharge was undermining the constitution, and, at the end of a month, Dr. C. determined to apply a ligature, for removal of the organ. On the 12th of February, therefore, the ligature (silk) was applied, and tightened two or three times a day. On the second day, the tumor was dark as a mulberry. Every day the ligature was tightened. On the sixth day, the sphacelated mass was removed by the knife below the ligature, which was, itself, removed on the eighth day. The health improved—the urine flowed spontaneously. By the 20th March, she had recovered, and was able to attend to her domestic occupations. It is now sixteen months since the operation, and no catamenia have, of course, appeared. She enjoys good health.

---

### Dr. Ramsbotham's Report on the Royal Maternity Charity. (From the Medical Gazette.)

During the year 1832, there were delivered in the eastern district of the Royal Maternity Charity, under the superintendence of Dr. Ramsbotham—

2,344 *women*—of which cases,

33 were twins—one in about every 71 cases: of these in 18 cases both heads presented; in 10 the presentations were head and breech, or inferior extremities —in 11 of those cases the children were both boys; in 11 both girls; and in 11 one girl and one boy. 1,197 were males—1,180 females—2,320 were presentations of some part of the head; of which 8 were face presentations—one in about every 297 births—51 were breech presentations, or of some part of the lower extremities—about one in every 46¾ births—of these 16 were twins—six were transverse presentations—about one in every 396 children. In two the placenta was entirely, and in one partially, implanted over the os uteri—one in every 781⅓ cases—11 were complicated with dangerous hæmorrhage before delivery, *not* the result of placental presentations—about one in every 213 cases. In 25 cases the placenta was retained within the uterus either by atony, irregular contraction, or morbid adhesion—two women were delivered by craniotomy—one in every 1,172 cases, in both the pelvis was narrowed very much. In four cases premature labour was induced—one in every 586 cases—in three by the use of ergot—in each the pelvis was contracted. The time chosen was between the 7th and 8th month of pregnancy—13 women died within the puerperal month, or from puerperal causes—one in every 180 cases—2,287 children born living—89 still-born, about one in every 25 births.

*Still-born Children.*—28 were premature—13 were putrid, at or near the full time—six breech presentations.

During the year 1833 there were delivered 2,619 women—of these cases

22 were twins—one in every 119 cases—1,372 males—1,269 females—2,437 head presentations—64 breech, or lower extremities—about one in every 41¼ births—of these 17 were twins. In 18 cases there was retention of the placenta —one in every 145½ cases—nine complicated with alarming hæmorrhage after expulsion of placenta, onei n every 291 cases—three were delivered by craniotomy, one in every 873 cases. In three premature labour was induced—five were complicated with puerperal convulsions, about one in every 524 cases—16 women died either from puerperal causes, or within the puerperal month—one in about every 166 cases—2,556 children were born living—85 still-born.

*Deaths.*—Four from hæmorrhages—one from puerperal convulsions—two from fever—one from diffused pelvic inflammation nine days after delivery by craniotomy—two from pneumonia—five from phthisis.

During the year 1834, there were delivered 2,447 women—of which cases 26 were twins, about one in every 94 cases—1,285 were males—1,188 females—2,408 were head-presentations—57 were breech or lower extremities—about one in every 43 births—eight were transverse—one in every 309 births—five had alarming accidental hæmorrhage, *not* the consequence of placental presentation—in 10 there was retention of placenta—6 had alarming hæmorrhage after placental expulsion—about one in 412 cases. Four were delivered by forceps—one in every 618¾ cases. Two had puerperal convulsions—in two premature labour was induced from contracted pelvis—one in every 1,236½ cases—10 women died within the puerperal month, about one in 245. 2,408 children were born living —79 still-born, about one in every 31⅓ births.

*Deaths.*—Three from hæmorrhage—one from convulsions—one from rupture of uterus—one from hysteritis—two from cholera—one from universal dropsy.

During the year 1835, 2,311 women were delivered in the Eastern District of the Royal Maternity Charity—of these cases 16 were twins—in 8 cases of these both hands presented; in 4 the presentation was head and breech or inferior extremities, and in one case the breech and shoulder presented. In 3 of those cases both children were males; in 6 both were females; and in 7 they were of different sexes—1,229 children were males—1,098 females—2,437 were head presentations—57 were breech or some part of the lower extremities—7 were transverse presentations—all of some part of the upper extremities. In 3 the placenta was implanted over the os uteri, that is, about 1 in every 776 cases.

Seven were complicated with alarming accidental hæmorrhage before delivery, *not* the result of placental presentation, that is, about 1 in every 330 cases. In 8 the placenta was retained within the uterus, either by irregular contraction or atony of the uterine fibres, or by morbid adhesion to the uterus—all required the introduction of the hand—5 were complicated with alarming hæmorrhage after the natural expulsion of the placenta—1 in every 462 cases. One (a primary labour) was delivered by craniotomy. Three were delivered by the forceps—about 1 in 776 cases. Four were complicated with puerperal convulsions—about 1 in every 578 cases. In all these cases the attack occurred during labour. All were benefited by bleeding and purging—they all recovered.

In one case premature labour was induced from the woman's possessing a small pelvis. Seven women died either from puerperal causes, or within the puerperal month, about 1 in every 330 cases. 2,241 children were born living —86 born dead—being about 1 in every 27 births.

*Of the Deaths.*—1 from the effects of hæmorrhage eleven days after labour—there had been a draining of blood for some weeks previously—2 after delivery under shoulder presentation, both on the third day—one after version, and one from hysteritis after decapitation. One on the eighth day after labour from apoplexy originating in violent mental agitation six days after labour. Two from malignant puerperal fever prevalent at the time—1 from peritonitis 31 days after delivery.

*Of Still-born Children.*—26 were premature—6 were putrid, at or nearly at their full time—16 were breech presentations, at or nearly at full time—4 were transverse—with 5 the funis prolapsed—one was delivered by craniotomy—1 by the forceps—2 under placental presentation—2 were acephalous.

During the year 1838, there were delivered in the Eastern District of the Royal Maternity Charity, 2,136 women, of which cases 25 were twins, that is about 1 in 85½ cases—1,115 were males, 1,046 females—2,087 head presentations—68 were presentations of the breech, or some part of the lower extremities—about 1 in 32 births—6 were tranverse, all of the shoulder or of some part of the upper extremities. In 1 the placenta was implanted over the os uteri—the breech presenting above—6 were complicated with alarming hæmorrhage before delivery, *not* the result of placental presentation—1 in every 356 cases—all the women recovered. In 11 cases the placenta was retained either by atony, irregular contraction, or morbid adhesion, about 1 in every 194 cases. Four were complicated with alarming hæmorrhage after the placenta was born, 1 in every 534 cases. Three were delivered by the forceps. In 1 premature labour was induced artificially at 7½ months from the woman's having but a small pelvis—2 were complicated with rupture of the uterus—9 women died either from puerperal causes, or within the puerperal month—2,076 children were born alive—85 children were still-born.

*Deaths.*—1 from hæmorrhage speedily after the birth of twins from injudicious extraction of the placenta—1 two hours after removing an adherent placenta—1 from hysteritis, 1 from collapse—2 from ruptured uterus—1 from puerperal mania, and 2 from common fever.

*Of the Still-born Children.*—19 premature—12 putrid at or near full time—14 were presentations of the breech or lower extremities—3 transverse—1 was delivered with the forceps: it was putrid—4 under lingering labour—2 under a ruptured uterus—2 under a face presentation—1 under a placental presentation—2 after violent accidental hæmorrhage—with 5 the funis prolapsed by the side of the head—with 7 the funis prolapsed by the side of the breech—1 was acephalous. Twelve were at full time, not putrid, nor delivered by art.

---

### Contributions to Midwifery, No. V. On the Influence of Ergot of Rye on the Fœtus in Utero. By Thomas Edward Beatty, M.D. M.R.I.A. (Condensed from the Dublin Journal of Medical Science, May 1844.)

Various and conflicting opinions have been entertained with respect to the influence of ergot of rye, as an obstetrical agent. Some very high authorities have declared that it is totally inefficient in exciting uterine action, under any circumstances. Other authorities, equally respectable, attribute the most energetic effects to its use, denouncing it as too violent an agent for obstetrical purposes, and as being injurious to the child at all times, and sometimes both to mother and child. A third class of authorities maintain an opinion equally at variance with the truth as the two preceding, viz. that the ergot may be given always with advantage, the safety of mother or child being never endangered. With respect to the first opinion, scil. that of its total inefficiency under any circumstances, Dr. Beatty says that two causes of such a failure may be suggested: 1, the employment of inadequate doses of the drug; 2, the inferior quality of that which was employed—the second cause appears the more probable, as there is scarcely any medicine that spoils more rapidly, or that requires more care in its preservation than the ergot.

The second opinion, viz. that the ergot of rye is at all times destructive to the child, arose from the employment of the medicine at improper times, as in cases of difficult labour arising from mechanical opposition to the exit of the child; in such a case the destruction of the child is almost sure to follow from the delay which necessarily occurs between the administration of the dose and the expulsion of the head. The third opinion, viz. that the ergot may be always given with advantage, is far too sweeping, and one likely to do mischief.

Dr. Beatty considers none of these opinions correct, but that the truth lies between them. The medicine, when fresh, and properly preserved, is one of great energy, and influences both mother and child. It requires to be used with great discretion; for, while it will in one case effect the delivery of a living child, it will, in another case, destroy the life of the child before birth, or operate so injuriously on it, as to cause its death shortly after birth, or produce a peculiarly dangerous effect on its nervous system. The difference of effect on the infant depends on the length of time intervening between the administration of the dose and the conclusion of the labour—if this takes place quickly, no harm is done to the child; if it be alive when the medicine is taken, it will be born so; but if a delay of even two hours should occur, the probability is, the child will be still-born. He here gives some cases wherein the duration of the labour after the administration of the medicine varied from a quarter of an hour to two hours, and in all the child was born without any unpleasant consequences.

In the cases which proved fatal, the condition of the infants was very unlike that of still-born children delivered under ordinary circumstances, and when no ergot had been administered to the mother. *The distinguishing characteristics are, the general lividity of the surface, the universal rigidity of the muscular system, producing the stiffened limbs and clenched hands in those infants in whom life was extinguished; and the remarkable kind of alternating spasm and palsy which supervened in those that were resuscitated.*

That the fœtus in utero may be influenced by the circulating fluids of the mother, is proved by the well-known fact of the communication of syphilis, small-pox, &c. to the unborn child.

Dr. B. here notices some of the epidemics of spasmodic ergotism, caused by eating bread made of rye containing a large portion of ergot, which visited different parts of the Continent during the last century. The patients were attacked with spasms and convulsions, accompanied with violent pains. In some instances the patients became lethargic, and when recovering from such state gave respectively signs of stupidity, intoxication, and extreme lassitude, after which the fit subsided for a time. There generally remained vertigo, *tinnitus aurium, nebulæ oculorum*, rigidity of the limbs, and great feebleness. It has been ascertained by actual experiment, that the blood of the mother becomes impregnated with the noxious properties of the ergot. In this way we can comprehend how the influence of the drug may be extended from the mother to the child. It may seem strange that a medicine taken in the usual medicinal doses, and with apparent impunity, by the mother, shall act injuriously on the child. The fact is, the system of the mother is very generally acted on by the ergot, though not injuriously, as is shewn by the great depression in the pulse, caused by the drug. We must also recollect the great susceptibility of infants to the action of narcotics.

From the preceding observations, Dr. Beatty comes to the conclusion, that the administration of ergot to a woman in labour is attended with danger to the child, whenever a time sufficient for the absorption and transmission of its noxious properties elapses before the child is born. The *degree* of effect produced differs with the time that elapses between the exhibition of the dose and the birth of the child. Hence, the ergot should never be given in any case where there is a likelihood of the labour lasting more than two hours after its administration, except when it may be employed to save the mother's life—and, 2dly, if delivery is delayed to two hours, we should resort to artificial assistance to save the life of the child.

